# The Solar Spectrum in the Atacama Desert

**DOI:** 10.1038/srep22457

**Published:** 2016-03-02

**Authors:** R. R. Cordero, A. Damiani, G. Seckmeyer, J. Jorquera, M. Caballero, P. Rowe, J. Ferrer, R. Mubarak, J. Carrasco, R. Rondanelli, M. Matus, D. Laroze

**Affiliations:** 1 Universidad de Santiago de Chile, Ave Bernardo O’Higgins 3363, Santiago, Chile; 2 Japan Agency for Marine-Earth Science and Technology, Yokohama, Japan; 3 Leibniz Universität Hannover, Herrenhäuser Str. 2, 30419 Hannover, Germany; 4 Universidad de Magallanes, Avenida Bulnes 01855, Punta Arenas, Chile; 5 Universidad de Chile, Blanco Encalada 2002, Santiago, Chile; 6 Center for Climate and Resilience Research (CR)2, Universidad de Chile, Santiago, Chile; 7 Universidad de Tarapacá, Casilla 7D, Arica, Chile

## Abstract

The Atacama Desert has been pointed out as one of the places on earth where the highest surface irradiance may occur. This area is characterized by its high altitude, prevalent cloudless conditions and relatively low columns of ozone and water vapor. Aimed at the characterization of the solar spectrum in the Atacama Desert, we carried out in February-March 2015 ground-based measurements of the spectral irradiance (from the ultraviolet to the near infrared) at seven locations that ranged from the city of Antofagasta (on the southern pacific coastline) to the Chajnantor Plateau (5,100 m altitude). Our spectral measurements allowed us to retrieve the total ozone column, the precipitable water, and the aerosol properties at each location. We found that changes in these parameters, as well as the shorter optical path length at high-altitude locations, lead to significant increases in the surface irradiance with the altitude. Our measurements show that, in the range 0–5100 m altitude, surface irradiance increases with the altitude by about 27% in the infrared range, 6% in the visible range, and 20% in the ultraviolet range. Spectral measurements carried out at the Izaña Observatory (Tenerife, Spain), in Hannover (Germany) and in Santiago (Chile), were used for further comparisons.

The surface solar spectrum depends on the solar zenith angle (SZA), cloudiness, aerosol concentrations, the columns of atmospheric constituents (such as ozone and water vapor), ground reflectivity (albedo), the Sun–Earth-distance and the altitude.

Since desert areas in subtropical continental regions are typically characterized by clear conditions^1^, the role of clouds on the solar irradiation tends to be less important than the effect of aerosols. Due to the dry and arid conditions, natural atmospheric aerosols (wind-blown dust) at desert locations significantly affect the surface irradiance. For example at sites in North Africa, the aerosol optical depth (AOD) is typically higher than 0.15 at 500 nm^2^. The AOD in the visible range measured at desert sites in northern China ranges from 0.24 to 0.36^3^. The AOD measured by a sunphotometer of the Aerosol Robotic Network (AERONET)^4^ in Arica (the northernmost Chilean city) during 2013 at 500 nm ranged from 0.1 to 0.3^5^. These values are slightly different than satellite-derived data corresponding to other locations in northern Chile. Figure 1a shows the average of AOD values computed by using daily SeaWIFS-derived estimates^6^ for February over the period 2003–2014. Note that the AOD values ranges from 0.1 to 0.3. Moreover, MODIS retrievals^7^,^8^,^9^ allow estimating climatological values of about 0.1 for the AOD at 550 nm in the Atacama Desert. These relatively low values of the AOD agree with the ground-based measurements carried out at the Paranal Observatory (2,635 m altitude, 24°37′S, 70°24′W)^10^.

The precipitable water (PW) column also affects the surface irradiance (especially in the IR part of the spectrum). MODIS-derived estimates of the PW over the period 2000–2009 show values lower than 5 mm over the Atacama Desert, the Tibetan Plateau, Greenland and Antarctica^11^. Figure 1b shows the average of PW values computed by using daily AIRS-derived estimates^12^ for February over the period 2003–2014. PW values lower than 5 mm are observed over the Andes. These relatively low values of the PW have been confirmed by ground-based measurements carried out at the Paranal Observatory^13^,^14^ and on the Chajnantor Plateau (5100 m altitude, 23°00′S, 67°45′W)^15^. Indeed, on the Chajnantor Plateau the amount of precipitable water is typically 1.0 mm and falls below 0.5 mm up to 25% of the time over the period May-November; during the “Bolivian Winter” (from the end of December to early April) the PW is slightly higher^15^.

The total ozone column (TOC) over the Atacama Desert is also low (when compared to locations in the northern hemisphere). Satellite estimates of the TOC value typically range in the Atacama Desert from 240 DU (in the austral summer) to 330 DU (in the austral winter)^16^. Figure 1c shows the average of TOC values computed by using daily AIRS-derived estimates of the TOC^17^ for February over the period 2003–2014. Average TOC values lower than 245 DU can be observed over the Andes. Mostly because the low TOC values registered in summer, the world’s highest levels of surface ultraviolet (UV) irradiance have been measured in the Atacama Desert. Indeed, ground-based measurements have shown the UV index (UVI), an international standard measure of the UV level that can lead to an erythemal or sunburning response in humans^18^,^19^, to exceed 20 in the Atacama Desert^20^,^21^.

The albedo of bright deserts affects the surface irradiance (especially in the UV part of the spectrum). Ground-based measurement of the spectral albedo of desert surfaces in western and central China^22^ have shown that the albedo in the UV part of the spectrum range from 0.05 to 0.11. These values agree with the data of Lambertian equivalent reflectivity (LER) recorded over the Atacama Desert in the UV-A range by the Ozone Monitoring Instrument (OMI) on EOS Aura^23^. Although the albedo increases with the wavelength up to about 2000 nm (slightly decreasing afterward^22^), the effect on the global irradiance of the relatively high albedo in the visible and IR tends to be small because of the low Rayleigh scattering at long wavelengths.

The earth is closer to the sun in the southern hemisphere summer compared with the corresponding season of the northern hemisphere. Only the effect the elliptical orbit of the earth around the sun, leads to about 7% difference in the interhemispherical peak irradiances^24^. Moreover, the different geographic distribution of the TOC, the PW, and the aerosols, further strengths these inter-hemispherical differences in the surface irradiance. Due to these factors, the highest surface irradiance is expected to occur in summer at high altitude sites in the SH near the Tropic of Capricorn, i.e. the Atacama Desert. Indeed, prior efforts based on available global datasets, a semi-empirical model and a network of pyranometers in northern Chile, have pointed the Atacama Desert as the place where the highest surface irradiance is likely to occur^11^.

The absolute solar spectral irradiance has many interests ranging from solar physics to climatology and Earth’s environment physics. Recent satellite-based measurements^25^,^26^ suggest a significantly stronger variability in the UV range and changes in the visible and infrared (IR) bands with respect to previous estimates, which may have implications on the Earth’s atmosphere. However, long and reliable time series of spectral irradiance measurements are scarce, which makes an accurate quantification of solar contributions to recent climate change difficult. In the particular case of the Atacama Desert, no spectral measurements in the visible and IR range have been reported; prior efforts targeted only the UV part of the spectrum^20^.

In what follows, we report on the first quality-controlled measurements of the solar spectrum in the Atacama Desert carried out using a double monochromator-based spectroradiometer. During a campaign conducted in February-March 2015, the spectral irradiance (from the UV to the near IR) was measured at seven locations that ranged from latitude 22°S to 28^o^, and from the city of Antofagasta (on the southern pacific coastline) to the Chajnantor Plateau (5,100 m altitude). Satellite estimates as well as spectral measurements carried out in Santiago de Chile (550 m above the sea level, 33°27′ S, 70°41′ W), at the Izaña Observatory (located on Tenerife island at 2367 m above sea level, 28°18′N, 16°30′W), and Hannover (50 m above sea level, 52°23′ N, 9°42′ E), were used for further comparisons.

## Measurements and Methods

### Ground-based measurements

Most instruments that can carry out spectral measurements are fast single-grating spectroradiometers fitted with CCD arrays (also known as polychromators)^22^,^27^,^28^. Although array instruments are capable of measuring the spectrum in a very short time interval, their measurements at short wavelengths (specifically in the UV-B) are strongly affected by stray light^29^,^30^,^31^,^32^,^33^. Intercomparisons involving several CCD arrays have shown significant differences in the measurements at wavelengths shorter than 350 nm^28^. Several ex-post stray light corrections have allowed improving the performance of array spectroradiometers in the UV range^31^. However, double monochromator-based spectroradiometers are still recommended for quality-controlled measurements of the surface irradiance (particularly in the UV part of the spectrum).

Double monochromator-based instruments developed according to the specifications defined by the World Meteorological Organization (WMO)^34^ and the Network for the Detection of Atmospheric Composition Change (NDACC)^35^ can produce measurements with uncertainties of up to 10% for UV-B wavelengths (290–315 nm) and up to 4% for UV-A wavelengths (315–400 nm)^32^,^36^. Uncertainties of spectral measurements in the visible (400–700 nm) and the IR (wavelengths longer than 700 nm) are expected to be similar (up to 4%) excepting around bands of strong water vapor absorption (1150 nm, 1400 nm, 1850 nm). Similar to what occurs with the radiation at wavelength shorter that 300 nm (strongly absorbed by the ozone), the relative uncertainty around 1150 nm, 1400 nm, and 1850 nm, tends to be greater because the strong absorption leads to a very weak signal (easily affected by the noise or the dark signal of the detector)^36^.

Spectral measurements reported below were carried out using a spectroradiometer system based on a double monochromator Bentham DTMS300, 300 mm focal length, fitted with a photomultiplier (PMT) detector (especially important in the UV range), a Silicon photodiode detector (suitable for spectral acquisitions up to about 1100 nm) and an InGaAs detector for wavelength longer that 1100 nm. The system has a set of gratings: a grating holographic of 2400 lines/mm (blazed at 250 nm) as well as gratings ruled 1200 lines/mm (blazed at 500 nm) and 600 lines/mm (blazed at 1200 nm). The Full With at Half Maximum (FWHM) of the spectroradiometer was 1 nm (in the range 290–650 nm) and 5 nm afterwards. The system was operated within a temperature-controlled weatherproof box. An integrating sphere fitted with a quartz dome was used as input optics for global irradiance. Although it is not an NDACC-certified instrument, our spectroradiometer complies with NDACC specifications^35^ and the WMO recommendations^34^.

### Satellite observations

Daytime ascending satellite observations of PW^12^ and TOC^17^ were retrieved from Atmospheric Infrared Sounder (AIRS) aboard NASA’s Aqua satellite. AIRS-derived climatological values were computed by using estimates over the period 2003–2014. AIRS is a high–spectral resolution infrared sounder with 2378 bands that measures outgoing radiances in the IR and 4 bands in the visible region of the spectrum. The field-of-view is 1.1° and the nominal spatial resolution is 13.5 km at nadir.

Satellite measurements of AOD at 550 nm have been retrieved from the Sea-viewing Wide Field-of-view Sensor (SeaWIFS), processed with the Deep Blue algorithm^6^. Although initially designed to measure ocean color, its measurements in the visible and in the infrared also provide information on atmospheric aerosols. In this paper we used the new SeaWiFS aerosol dataset for 1997–2010 that has been produced as part of NASA’s MEaSUREs project^37^.

### Field Campaign

During a field campaign in February-March 2015, the solar spectral irradiance was measured at seven locations in the Atacama desert (see Table 1), ranging from latitude 22^o^S to 28^o^, and from Antofagasta City (on the southern pacific coastline) to the Chajnantor Plateau (5,100 m altitude). Measurements were made using a double monochromator-based spectroradiometer described above.

The spectroradiometer sampled the irradiance every 1 nm (in the range 290–650 nm) and every 5 nm afterwards; scans were carried at each location at a 60 min interval. Dates and conditions during the campaign are indicated in Table 1. The absolute calibration of the spectroradiometer was achieved using a field calibrator fitted with a baffled 150 W quartz halogen lamp. Based on the certificate of the lamp and the transfer of calibrations, we estimated the uncertainty involved in the absolute calibration to be up to 10% for UV-B wavelengths and up to 4% for longer wavelengths (excepting around bands of strong water vapor absorption (1150 nm, 1400 nm, 1850 nm).

The UV-B, UV-A, visible, and IR irradiances, computed by integrating the measured spectra, have uncertainties similar to the spectral measurements within the corresponding interval of integration^36^. For example, the visible irradiance computed by integrating the spectral irradiance from 400 to 700 nm is expected to be about 4%. By comparison, uncertainties in the range 7–16% have been reported for UV measurements made by radiometers^38^,^39^ and uncertainties in the range 3% to 5% have been reported for pyranometers^40^.

### Data Exploitation

The ground-based based spectral measurements of solar irradiance were exploited in order to retrieve the total ozone column (TOC), the precipitable water (PW), and the parameters that represent the aerosol influence: the aerosol optical depth (AOD) and the single scattering albedo (SSA).

Under cloudless conditions, the TOC values were retrieved from our measurements of the spectral irradiance by applying a method that implied comparing the ratio (between irradiances measured at UV-B wavelengths and at UV-A wavelengths) with a synthetic chart of this ratio computed for a variety of TOC values^41^. In a similar way, the PW values were also computed from our measurements under cloudless conditions by comparing the ratio between irradiances measured at different wavelengths (around the absorption band of water vapor at 945 nm) with a synthetic chart of this ratio computed for a variety of PW values^42^.

In order to retrieve the parameters that represent the aerosol influence, we carried out at each location a limited number of spectral measurements of both the global and the diffuse irradiance (by using a shadow ring). These measurements (performed at each location around noon) allowed us to retrieve both AOD and the SSA by applying the methods described elsewhere in the case of the AOD^43^, and in the case of the SSA^44^,^45^. These methods are based on the comparison of the measured spectral irradiance with spectra computed by using a radiative transfer model. The retrieved values of the AOD and SSA are those leading to the best match between the measured and the computed spectra.

The radiative transfer model used was the UVSPEC model, which is the main tool of the libRadtran package for radiative transfer calculations^46^. In the UV spectral range, this model has been validated by systematic comparisons with ground-based measurements under cloudless conditions in other geographic regions^47^,^48^. The model used the DIScrete Ordinates Radiative Transfer (DISORT) solver^49^ and the extraterrestrial spectrum of Gueymard^50^. Although it is difficult to characterize the cloud effect with such models, they are useful for checking the consistency of surface measurements.

## Results

### Spectral Irradiance

Figure 2 shows samples of spectra measured at each of the seven locations indicated in Table 1. Dates (day.month.year) are indicated in the plots while the color of the spectra indicates the local time (LT) of the measurement. The main absorption lines (corresponding to O_3_, O_2_ and H_2_O) are indicated in the plots. Differences are apparent between the spectra. For example, the depths of the bands of water vapor absorption around 945 nm, 1150 nm and 1400 nm, are clearly different between the spectra measured in Antofagasta (see Fig. 2a) and on the Chajnantor Plateau (see Fig. 2g). As shown below, the relatively low water vapor absorption on the Chajnantor Plateau is associated with the very low PW at that location.

As a test of self-consistency, the AOD, SSA, PW, and TOC derived from the measured spectra around noon (shortly before 14:00 LT) at each location, were used as inputs to the UVSPEC/LibRadtran model. The simulated spectra (black dashed lines) are also shown in Fig. 2 but, due to the goodness of agreement, are generally obscured by the measured spectra at 14:00 LT (red solid lines) that are plotted over them. Indeed, measured and computed spectra generally agree within the bounds defined by the uncertainties of our measurements: up to 10% for UV-B wavelengths and up to 4% for longer wavelengths (excepting around bands of strong water vapor absorption (1150 nm, 1400 nm, 1850 nm).

### Irradiance

Differences in the irradiances (IR, visible, UV-A and UV-B irradiances), computed by integrating the spectra measured at different locations are expected due to the obvious differences between the spectra shown in Fig. 2. For example, the spectral irradiances measured at 14:00 LT on the Chajnantor Plateau are noticeably higher than the spectral irradiancies measured at 14:00 LT in Antofagasta (see red curves in Fig. 2a,g).

Although the plots in Fig. 2 allow quick comparisons between the irradiances at each location, a systematic assessment requires comparing irradiances at common solar zenith angles (SZAs). In order to facilitate such comparisons, the measured spectra were linearly interpolated to common SZAs and the ratio was taken between the irradiances at different locations and the irradiance in Antofagasta on 08.02.2015 (see Fig. 3).

Figure 3a (black line) shows that the UV irradiance on the Chajnantor Plateau (5100 m altitude) is, regardless of the SZA, about 20% higher than the UV irradiance in Antofagasta (114 m altitude). Differences even greater (about 27%) are shown in Fig. 3c (black line) between the IR irradiance on the Chajnantor Plateau and in Antofagasta. These values are significantly greater than the ratios of visible irradiances, which as shown in Fig. 3b, were always lower than 10%.

The differences in the irradiances shown in Fig. 3 are expected due to the differences in altitude of the seven locations. Indeed, the differences are significantly smaller between locations at closer altitudes. For example, it can be seen in Fig. 3a,c, that the differences between irradiances in El Salvador (1600 m altitude; see green line) and in Calama (2293 m altitude; see purple line) are within the bounds defined by the uncertainties of our measurements. The same is true for the irradiances in Caldera (208 m altitude; see blue line) versus Antofagasta (114 m altitude), as well as the irradiances in M. Elena (1184 m altitude; see brown line) and in D. de Almagro (780 m altitude; see orange line).

Figure 4 illustrates the general increase in irradiance with altitude. Dots indicate the average of the irradiance measured at each location at SZA = 20°. Although there is not enough data for carrying out a robust statistical analysis, by comparing the irradiances at different altitudes, we were able to roughly estimate the increases with altitude (see Table 2). For example, IR irradiance increased with altitude by about 5.5% per km over the range 0–2500 m altitude and around 3.5% per km over the range 2500–5000 m altitude. The increases in irradiances with the altitude shown in Table 2 were used to create the dashed blue lines shown in Fig. 4. However, as discussed below, local conditions (e.g. SSA, AOD, PW) may change not only with the altitude but also with time (exhibiting daily and seasonal variations). Therefore, actual increases in irradiance with altitude will vary from those shown in Table 2.

As shown in Table 2, the greatest increase with the altitude was observed in the case of the UV-B irradiance (calculated by integrating the spectra over the interval 290–315 nm). We found an increment in the UV-B irradiance of about 9% per km over the range 0–2500 m altitude and around 4% per km over the range 2500–5000 m altitude. The increment of about 4% per km in the UV-B over the range 2500–5000 m altitude agrees with the increment in the UV index reported elsewhere^20^ using measurements carried out on the Chajnantor Plateau and at the Paranal Observatory (2635 m altitude, 24°37′S, 70°24′W). The increase in UV-B irradiance with height in the range 0–2500 m (about 9%) is similar to those found elsewhere: 7% per km in the Himalayas^51^; 6.5% per km in Hawaii^52^; and 9% per km in the Alps^53^.

### Ozone and Water Vapor

Figure 5a,b show the retrievals of the TOC and PW derived from the spectra measured at different times during the campaign. The differences between the retrieved values of the TOC at different locations (see Fig. 5a) were not as marked as those detected between the retrieved values of the PW at different locations (see Fig. 5b).

**Figure 1:**
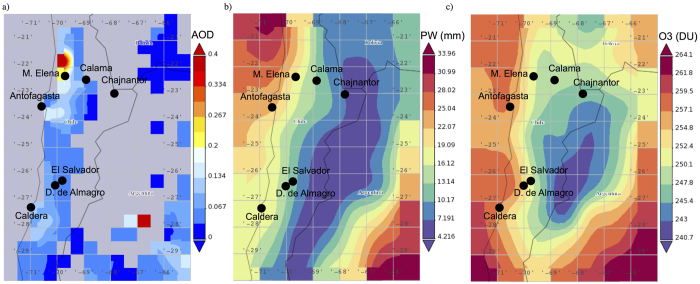
(**a**) Average of AOD values computed by using daily SeaWIFS-derived estimates for February over the period 1997–2010. Maps based on gridded observations with a 0.5° × 0.5° resolution. The seven locations where we carried out ground-based measurements are also shown. (**b**) Average of PW values computed by using daily AIRS-derived estimates for February over the period 2003–2014. Maps based on level-3 gridded observations with a 1° × 1° resolution. (**c)** Average of TOC values computed by using daily AIRS-derived estimates for February over the period 2003–2014. Maps based on level-3 gridded observations with a 1° × 1° resolution. Maps produced by using the Giovanni online data system (version 4.15), developed and maintained by the NASA GES DISC. Available at: http://giovanni.gsfc.nasa.gov/giovanni/. (Accessed: 4th June 2015).

**Figure 2:**
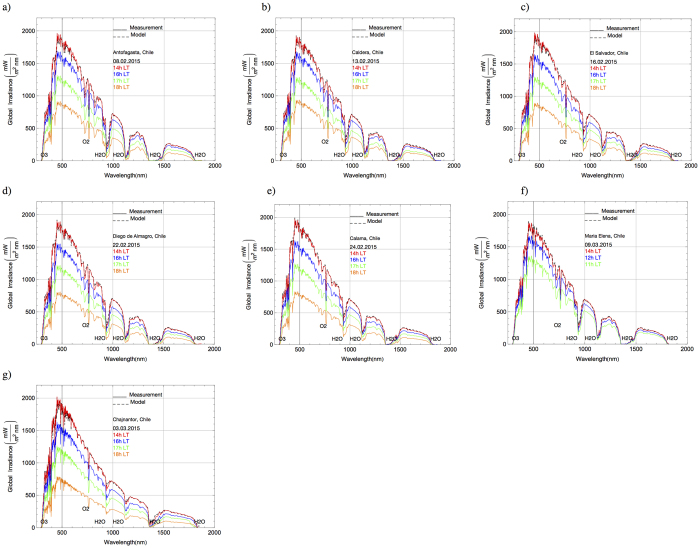
Solid lines correspond to spectra measured at each location. Dates (day.month.year) are indicated in the plots. Color indicates the local time (LT). Black dashed lines correspond to spectra yielded by the UVSPEC/LibRadtran radiative transfer model under the conditions observed at each location at 14:00 h LT. The main absorption lines are indicated in the plots. (**a**) Antofagasta; (**b**) Caldera; (**c**) El Salvador; (**d**) Diego de Almagro; (**e**) Calama; (**f**) Maria Elena; (**g**) Chajnantor.

**Figure 3:**
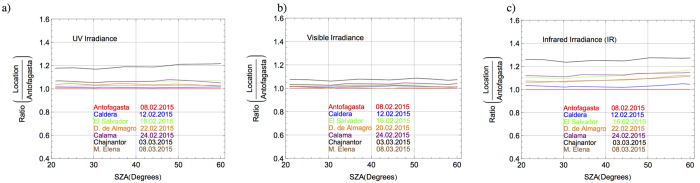
Ratio between the irradiances at each location and the irradiance in Antofagasta. The irradiances are compared at the same solar zenith angles (SZA). Color indicates the location. Dates (day.month.year) are indicated in the plots. (**a**) UV irradiance (calculated by integrating the measured spectra over the interval 290–400 nm). (**b**) Visible irradiance (calculated by integrating the measured spectra over the interval 400–700 nm). (**c**) Infrared irradiance (calculated by integrating the measured spectra at wavelengths longer than 700 nm).

**Figure 4:**
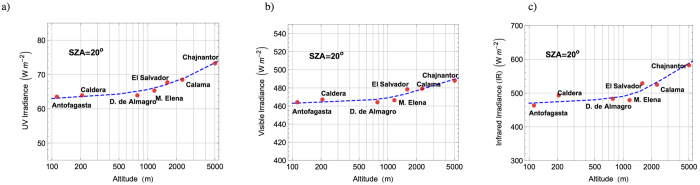
Dots stand for the average of the irradiance (calculated by integrating the spectra measured at SZA = 20°).The dashed line was created by using the increases with the altitude shown in Table 2. (**a**) UV irradiance. (**b**) Visible irradiance. (**c**) Infrared irradiance.

According to satellite estimates (see Fig. 1c), a difference in the ozone column of a few Dobson Units (DU) among sites located at different altitudes was expected. Actually, this is the basic assumption of the Topographic Contrast Method for deriving tropospheric ozone from satellite observations^54^. However, the day-to-day ozone variability during the campaign partially masked the expected changes in the TOC with altitude. Indeed, the TOC values exhibited differences between consecutive days similar to those between different locations. For example, the TOC changed in El Salvador from 240 DU on 16.02.2015 to 245 DU on 17.02.2015. This difference (5 DU) is similar to that found when comparing the TOC on the Chajnantor Plateau on 02.03.2015 (252 DU) and the TOC retrieved in Caldera on 13.02.2015 (247 DU).

The PW significantly changed between consecutive days (for example, PW changed in Calama from 7 mm on 25.02.2015 to 15 mm on 26.02.2015). However, as shown by satellite estimates (see Fig. 1b), we measured significantly low PW values at high altitude locations. For example, the PW was 35 mm at 14:00 in Antofagasta on 08.02.2015 but less than 2 mm on the Chajnantor Plateau on 03.03.2015.

**Figure 5:**
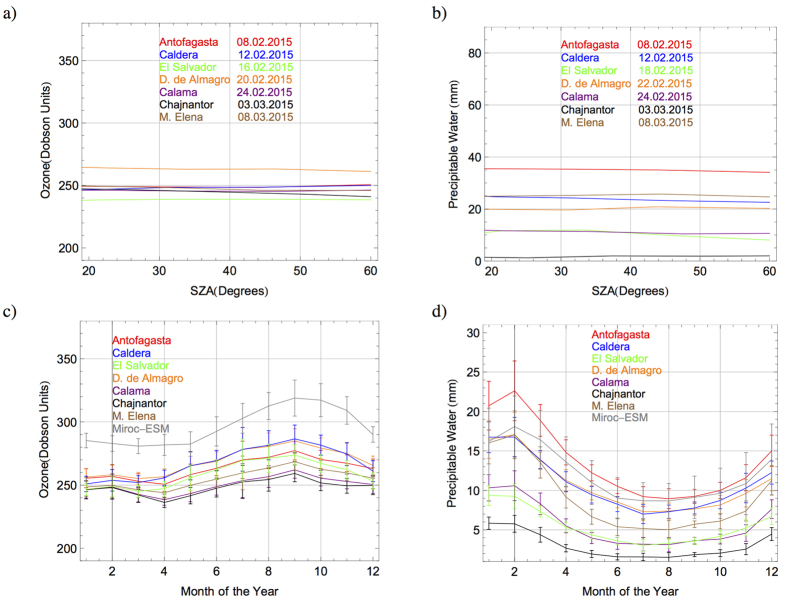
(**a**) Total Ozone Column (TOC) (color indicates the location) derived from the spectra measured at different times through the day during the campaign. Dates (day.month.year) are indicated in the plots. (**b**) Precipitable Water (PW) (color indicates the location) derived from the spectra measured at different times through the day during the campaign. Dates (day.month.year) are indicated in the plots. (**c**) Monthly climatology of TOC. Solid lines indicate the averages of the TOC values (color indicates the location) computed by using daily AIRS-derived estimates over the period 2003–2014. The TOC climatology for years 1996–2005 from the MIROC–ESM-CHEM model is also shown. Bars indicate the observed variability taken as equal to the standard deviation of the series of monthly averages. (**d**) Monthly climatology of PW. Solid lines indicate the averages of the PW values (color indicates the location) computed by using daily AIRS-derived estimates over the period 2003–2014. The PW climatology for years 1996–2005 from the MIROC-ESM-CHEM model is also shown. Bars indicate the observed variability taken as equal to the standard deviation of the series of monthly averages.

For further comparisons, Fig. 5c shows time series of the climatological annual cycle of monthly TOC during the 2003–2014 period as retrieved from AIRS observations for the different locations examined here. The TOC values measured in February/March (see Fig. 5a) and the satellite-derived climatological values (see Fig. 5c) generally agree within the bounds defined by the variability of the satellite readings; bars in Fig. 5c indicate the observed variability taken as equal to the standard deviation of the series of monthly averages. Figure 5c shows that, overall, the amplitude of the annual cycle of the TOC is about 20–30 DU with higher values in September and lower values in summer and early autumn. The TOC climatology for years 1996–2005 from the MIROC-ESM-CHEM model^55^ averaged over the region of interest is also shown in Fig. 5c. Although biased high, MIROC-ESM-CHEM reproduces the annual cycle reveled by satellite data.

Figure 5d shows time series of the climatological annual cycle of monthly PW during the 2003–2014 period as retrieved from AIRS observations. The PW values measured in February/March (see Fig. 5b) and the satellite-derived climatological values (see Fig. 5d) generally agree within the bounds defined by the variability of the satellite readings. Figure 5d also shows that the PW displays a seasonal cycle peaking generally in February while winter is characterized by lower PW values. Moreover, in agreement with our measurements, in Fig. 5d high amounts of PW are evident near the coastline (e.g. Antofagasta) while very low values are evident in the interior (e.g. Chajnantor). The estimates by MIROC-ESM-CHEM model, averaged over the region of interest (also shown in Fig. 5d), capture both the shape and amplitude of the PW annual cycle reveled by satellite data.

## Discussion

The differences between the surface irradiances described above between locations in the Atacama Desert are mostly due to differences in the altitude, in the aerosol loading, and in the TOC and PW values. In what follows, the effect of these differences are characterized and discussed.

### Effect of Ozone

**Figure 6:**
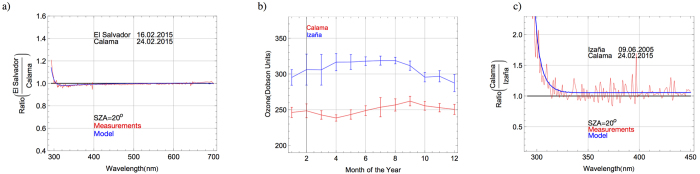
(**a**) Ratio between the solar spectra (at SZA = 20°) in El Salvador and in Calama. Dates (day.month.year) are indicated in the plots. (**b**) Monthly climatology of the TOC values in Izaña (blue line) and in Calama (red line) computed by using daily AIRS-derived estimates over the period 2003–2014. Bars indicate the observed variability taken as equal to the standard deviation of the series of monthly averages. (**c**) Ratio between the solar spectra (at SZA = 20°) in Calama and in Izaña. Dates (day.month.year) are indicated in the plots.

Although large regional variations in ozone were not detected by our measurements (see Fig. 5a), relatively small differences in the TOC lead to significant changes in the UV spectrum (particularly in the UV-B part of the spectrum). Figure 6a shows the ratio (in the range 290–700 nm) between the solar spectra (at SZA = 20°) in El Salvador (on 16.02.2015) and in Calama (on 24.02.2015); red line in Fig. 6a stands for the ratio between measured spectra while the blue line in Fig. 6a represents the ratio between modeled (by the UVSPEC/LibRadtran model) spectra. Although Calama is at a slightly higher altitude than El Salvador (2293 m versus 1600 m), we detected similar aerosol loading and water vapor columns on 16.02.2015 in El Salvador, and on 24.02.2015 in Calama. Therefore, the ratio between the spectra at these locations (see Fig. 6a) is very close to 1, except in the UV-B part of the spectrum.

The differences depicted in Fig. 6a, between the spectra measured in Calama and in El Salvador at short wavelengths (<300 nm) are largely due to the differences in the TOC values (240 DU in El Salvador versus 247 DU in Calama). Due to differences in TOC, the spectral irradiance at around 300 nm in El Salvador is about 5% higher than the spectral irradiance in Calama (despite the fact that Calama is at a higher altitude than El Salvador). Greater relative differences can be observed in Fig. 6a at shorter wavelengths. Although UV-B irradiance represents a very small fraction (about 0.2–0.3%) of the total irradiance, it may lead to adverse effects on the biosphere including terrestrial and aquatic ecosystems and public health^19^, as well as to accelerated degradation of photovoltaic (PV) technologies^56^,^57^,^58^. Therefore, its characterization is required.

The Atacama Desert is characterized by relatively low TOC values. Figure 6b shows the monthly climatology of TOC for 2003–2014 retrieved from AIRS observations over Calama (red line) and at the Izaña Observatory (blue line); both locations are roughly at the same altitude. Seasonal variations in the ozone values are apparent. Monthly averages in Calama peak in austral spring. Moreover, peak ozone values are higher at the Izaña Observatory than in Calama. The average of the AIRS-derived estimates of the TOC at the Izaña Observatory in June (318 DU) is typically about 28% higher than in Calama in February (248 DU). The effect of these hemispherical differences in ozone can be better understood by comparing spectra measured in different hemispheres.

Figure 6c shows the ratio (in the range 290–450 nm) between the solar spectra at SZA = 20°, in Calama (on 24.02.2015, during the austral summer) and at the Izaña Observatory (on 09.06.2005, during the boreal spring). Red line in Fig. 6c stands for the ratio between measured spectra while the blue line in Fig. 6c represents the ratio between modeled (by the UVSPEC/LibRadtran model) spectra. Similarly that in the case of Calama, the spectrum at the Izaña Observatory was measured using a double monochromator-based spectroradiometer set up with a FWHM = 0.5 nm. The different spectral resolution of our instrument (in the case of Calama, the instrument FWHM was 1 nm) led to some *high frequency* artifacts in the ratio between the measured spectra (see red line in Fig. 6c). These artifacts are due to instruments features. Since they are not meaningful, they are not present in the ratio between the modeled spectra (see blue line in Fig. 6c). For the modeled spectrum in the case of the Izaña Observatory, the parameters used to represent the aerosol influence are available since this observatory is a contributor of AERONET^4^. On 09.06.2005, the SSA at Izaña Observatory was 0.94 and the AOD was 0.05 at 550 nm, which are similar to those detected in Calama on 24.02.2015.

At wavelengths longer than 330 nm, the differences shown in Fig. 6c (about 5%) in the spectral irradiances are mainly due to the different Sun–Earth distance (the Earth is closer to the Sun in the austral summer than in the boreal spring). Indeed, this individual factor leads to differences in irradiance of up to 7% between austral summer and boreal summer. However, much larger differences exist in the UV-B range. Figure 6c shows that at 300 nm, the irradiance in Calama is 100% greater than the irradiance at the Izaña Observatory. These extreme differences are due to the hemispherical differences in TOC values (see Fig. 6b). The TOC value on 09.06.2005 was at Izaña Observatory 310 DU, while in Calama was on 24.02.2015 only 247 DU. This difference in the ozone also leads differences of about 20% in the UV-B irradiance (calculated by integrating the measured spectra over the interval 290–315 nm).

### Effect of Water Vapor

**Figure 7:**
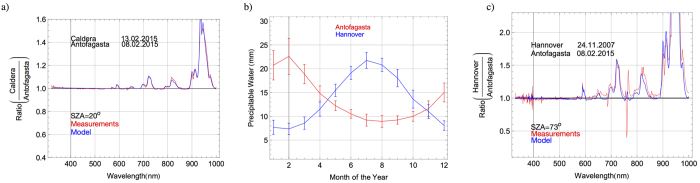
(**a**) Ratio between the solar spectra (at SZA = 20°) in Caldera and in Antofagasta. Dates (day.month.year) are indicated in the plots. (**b**) Monthly climatology of the PW values in Antofagasta (blue line) and in Hannover (red line) computed by using daily AIRS-derived estimates over the period 2003–2014. Bars indicate the observed variability taken as equal to the standard deviation of the series of monthly averages. (**c**) Ratio between the solar spectra (at SZA = 73^o^) in Hannover and in Antofagasta. Dates (day.month.year) are indicated in the plots.

As discussed above, we detected not only regional differences in PW within the Atacama Desert (see Fig. 5b) but also changes between consecutive days. These differences are relevant because they have significant effects on the spectrum, particularly in the IR. Figure 7a shows the ratio (in the range 315–1050 nm) between the solar spectra in Caldera (on 13.02.2015) and in Antofagasta (on 08.02.2015). Red line in Fig. 7a stands for the ratio between measured spectra while the blue line in Fig. 7a represents the ratio between modeled (by the UVSPEC/LibRadtran model) spectra. These two locations have similar altitudes (see Table 1), and exhibited similar aerosol loadings during the campaign. Moreover, comparable ozone columns were detected on 08.02.2015 in Antofagasta and on 13.02.2015 in Caldera. Therefore, the ratio between the spectra at these locations (see Fig. 7a) is very close to 1 except around the absorption bands of water vapor. Indeed, due to the different PW values (35 mm in Antofagasta versus 18 mm in Caldera), it can be observed in Fig. 7a that at around 945 nm, the spectral irradiance in Caldera is up to 60% greater than the spectral irradiance in Antofagasta. Greater relative differences were found around other absorption bands of water vapor (1150 nm, 1400 nm, 1850 nm).

Although differences in the PW values lead to significant effects on the spectrum, as shown in Fig. 7a, these differences are concentrated mainly in the infrared part of the spectrum. This is why, although the irradiance in Caldera is much higher than in Antofagasta at certain wavelengths, the difference in total irradiance is only about 3% (990 W/m^2^ in Antofagasta versus 1022 W/m^2^ in Caldera, at SZA = 20°). Nevertheless, even relatively small gains in the irradiance may be relevant in the case of utility-scale solar power plants.

MODIS-derived estimates of PW over the period 2000–2009 are very low over the Atacama Desert^11^. Record low values of the PW have been measured at the Paranal Observatory^13^,^14^ and on the Chajnantor Plateau^15^. Therefore, the Atacama Desert is generally considered a zone with a low water vapor column. However, as shown previously in Fig. 5b, there are significant regional differences within the Atacama Desert, such that the PW values in Antofagasta (on the southern pacific coastline) are actually high. For example, Fig. 7b compares the AIRS-derived monthly climatology of PW values over the period 2003–2014 in Antofagasta (see red line) to Hannover, Germany (see blue line). Despite the seasonal variations, it can be observed in Fig. 7b that the PW values tend to be lower in November in Hannover than in February in Antofagasta. The effect of these differences becomes apparent comparing spectra measured in these cities.

Figure 7c shows the ratio (in the range 315–1050 nm) between the solar spectra at SZA = 73^o^ in Antofagasta (on 08.02.2015, during the austral summer) and in Hannover (on 24.11.2007, during the boreal fall). Red line in Fig. 7c stands for the ratio between measured spectra while the blue line in Fig. 7c represents the ratio between modeled (by the UVSPEC/LibRadtran model) spectra. The spectrum in Hannover was also measured using a double monochromator-based spectroradiometer set up with a FWHM = 1 nm at 300–500 nm wavelength range, and a FWHM = 2 nm at 500–1050 nm wavelength range. As shown previously in Fig. 6c, the different spectral resolution of our instrument (in Antofagasta, the instrument FWHM was 1 nm up to 650 nm wavelength, and a FWHM = 5 nm afterwards) led to some meaningless high frequency artifacts in the ratio in Fig. 7c (see red line) that do not appear in the ratio between the modeled spectra (see blue line).

Hannover and Antofagasta have similar altitudes (50 m in Hannover versus 114 m in Antofagasta) and because of the dates, the role of difference in the earth-sun distance is presumably minor. In the UV-A and visible ranges, small differences (about 3% at wavelengths shorter than 500 nm) can be observed between the compared spectra in Fig. 7c. These differences are due to the aerosol loading. Although the AOD at 550 nm at both locations was comparable at the time of the measurements (0.11 in Hannover versus 0.12 in Antofagasta), the different composition of aerosols, i.e. the SSA (0.8 in Hannover versus 0.9 in Antofagasta) explains most of the differences in the UV-A and visible range. In the IR range, the differences are due to the different PW values (5 mm in Hannover versus 35 mm in Antofagasta).

### Effect of Aerosols

**Figure 8:**
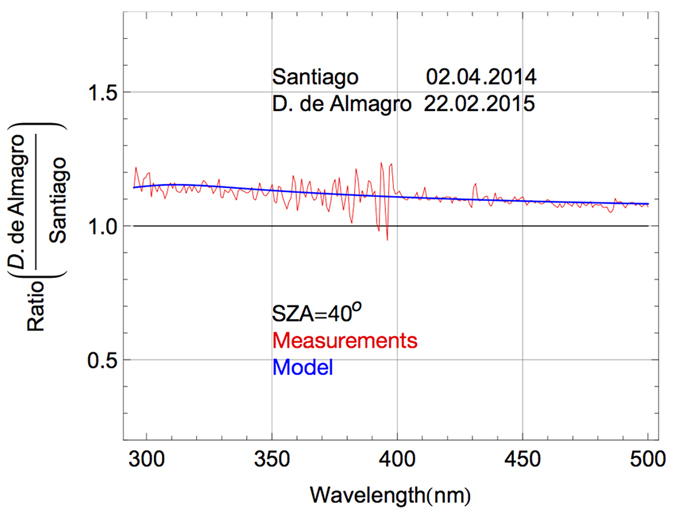
Ratio between the solar spectra (at SZA = 40^o^) in D. de Almagro (780 m altitude) and in Santiago de Chile (550 m altitude).

Aerosol loadings at each of the locations during the campaign were found to be low, particularly on the Chajnantor Plateau. Therefore, aerosol differences explain relatively little of the differences between the spectra measured during the campaign. Figure 8 demonstrates the effect on the local spectrum of the aerosol load in the Atacama Desert. It shows the ratio (in the range 290–500 nm) between the spectra (at SZA = 40°) in D. de Almagro (on 22.02.2015) and in Santiago de Chile (on 22.04.2014). Red line in Fig. 8 stands for the ratio between measured spectra while the blue line in Fig. 8 represents the ratio between modeled (by the UVSPEC/LibRadtran model) spectra. Santiago de Chile is a mid-latitude city of 6 million inhabitants with a complicated surrounding topography^59^, heavily affected by urban pollution^60^. The spectrum in Santiago de Chile was measured using a double monochromator-based spectroradiometer set up with a FWHM equal to 1.5 nm at 290–500 nm wavelength range. As explained above, the different spectral resolution of our instrument led to some high frequency artifacts in the ratio between the measured spectra in Fig. 8 (see red line) which do not appear in the ratio between the modeled spectra (see blue line).

Santiago de Chile and D. de Almagro have similar altitudes (550 m in Santiago de Chile versus 780 m in D. de Almagro). The ozone columns were also the same (about 252 DU) at the two locations at the moment of the measurements. Therefore, the differences observed in the UV and in the visible range in Fig. 8 are due to differences in the aerosols. Although the AOD at 550 nm at both locations was comparable at the time of the measurements (0.15 in Santiago de Chile versus 0.12 in D. de Almagro), the different compositions of aerosols led to very different SSAs (0.7 in Santiago de Chile versus 0.9 in D. de Almagro), which explains most of the difference between the spectra (i.e. the non-unit ratio in Fig. 8). Model spectra indicate that differences in the aerosol such as these may lead to differences of up to 7% in total irradiance (5% in the infrared range, 8% in the visible irradiance, 13% in the UV-A irradiance and up to 15% in the UV-B irradiance).

### Effect of Altitude

Changes in the spectrum with altitude are expected since higher altitude corresponds to a shorter path length through the atmosphere^61^. Moreover, satellite-derived estimates show that increases in altitude are associated with decreases in the column amounts of ozone, water vapor and other absorbers and scatterers, including aerosols.

**Figure 9:**
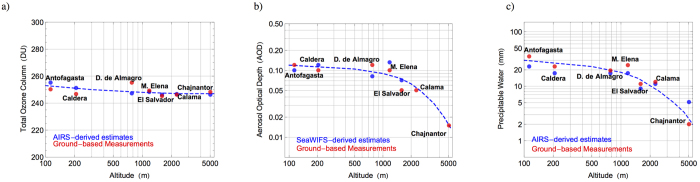
Average of our ground-based measurements compared with the satellite-derived climatological values for February. (**a**) Total Ozone Column (TOC). (**b**) Aerosol Optical Depth (AOD) at 550 nm wavelength. (**c**) Precipitable Water (PW).

The red dots in Fig. 9 show the averages of the TOC values (Fig. 9a), of the AOD values (Fig. 9b), and of the PW values (Fig. 9c), retrieved from our spectral measurements at different locations in the Atacama Desert. The blue dots in Fig. 9 show the satellite-derived climatological values for February at the same locations.

It can be observed in Fig. 9a that, although the daily variability partially masked the expected changes with altitude in our ozone measurements, satellite-retrieved data do show that the TOC reduces as the altitude increases in the Atacama Desert. Changes in the aerosol loading as well as in the PW amount with altitude were detectable by our ground-based measurements. In the case of the AOD (see Fig. 9b), SeaWIFS-derived data are not available for February over locations at altitude greater than 2500 m (Calama and Chajanantor). The satellite-derived climatological data at the rest of the locations do not clearly show changes with the altitude. However, our ground-based measurements did show that the AOD reduced as the altitude increased. The aerosol composition also changed with altitude such that greater values of the SSA were detected at higher locations, ranging from 0.90 in Antofagasta to 0.98 on the Chajnantor Plateau. Finally, as shown in Fig. 9c, both satellite-retrieved data and ground-based measurements indicate that relatively low PW values are associated with high-altitude locations.

**Figure 10:**
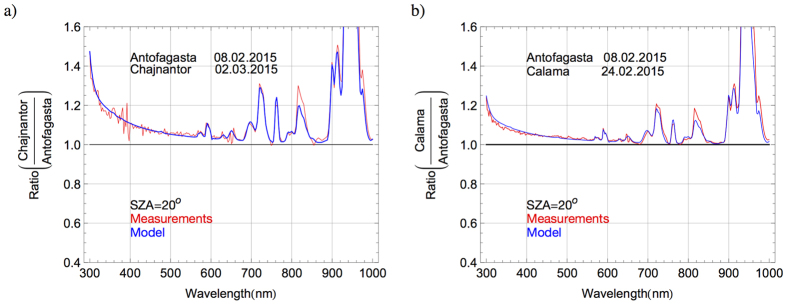
(**a**) Ratio between the solar spectra (at SZA = 20°) on the Chajnantor Plateau and in Antofagasta. (**b**) Ratio between the solar spectra (at SZA = 20°) in Calama and in Antofagasta.

Figure 10a displays the ratio of spectra on the Chajnantor Plateau (on 02.03.2015) and in Antofagasta (on 08.02.2015) in the range 300–1050 nm, while Fig. 10b shows the ratio of the spectra in Calama (on 24.02.2015) and in Antofagasta (on 08.02.2015). Red line in Fig. 10 stands for the ratio between measured spectra while the blue line in Fig. 10 represents the ratio between modeled (by the UVSPEC/LibRadtran model) spectra.

As shown in Fig. 10, the irradiances are significantly greater on the Chajnantor Plateau and in Calama than in Antofagasta. In the IR range differences arise due to the differences in the columns of some absorbers (in particular the water vapor and the oxygen). For example, the oxygen column decreases with altitude, and this explains the significant differences observed in Fig. 10a,b around 760 nm (corresponding to the oxygen absorption band). Significant differences can be also observed in Fig. 10a,b around the bands of water vapor absorption. This is due to the fact that, as shown in Fig. 9c, the PW tends to be low at high-altitude locations.

In the visible and UV ranges, Fig. 10a,b illustrate that differences between the compared spectra tend to decrease as the wavelength lengthens. In Fig. 10a for example, the irradiance (at 300 nm wavelength) on the Chajnantor Plateau is about 30% higher than the irradiance in Antofagasta. The difference between the spectra reduces to less than 10% at 500 nm wavelength. The wavelength-dependent difference between the spectra on the Chajnantor Plateau and in Antofagasta arises from the differences in the path length through scatterers. Since the scattering is greater at shorter wavelengths, the relative effect of the reduction in the path length is higher at shorter wavelengths.

The difference in the aerosol loading (that changed with the altitude; see Fig. 9b) also significantly contributes to the differences shown in Fig. 10a,b, especially in the visible and UV range. Actually, the differences in the UV are mostly due to the influence of the differences in the aerosol loading and in the path length through scatterers. The effect of the ozone in Fig. 10a,b is negligible since the TOC was nearly the same at those locations at the moment of the measurements.

### Disentangling the Effects

**Figure 11:**
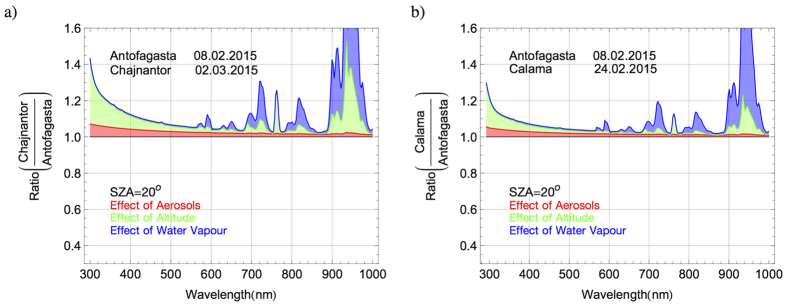
(**a**) Ratio between the solar spectra modeled (at SZA = 20°) assuming the condition observed (see dates in the plot) on the Chajnantor Plateau and in Antofagasta. The shaded areas indicate the effects of the different aerosol loading (red area), the shorter optical path length (green area), and the different Precipitable Water (PW) (blue area). (**b**) Ratio between the solar spectra modeled (at SZA = 20°) assuming the condition observed (see dates in the plot) in Calama and in Antofagasta. The shaded areas indicate the effects of the different aerosol loading (red area), the shorter optical path length (green area), and the different Precipitable Water (PW) (blue area).

Disentangling the influences of aerosols, PW, and altitude on the differences shown in Fig. 10a,b requires applying a radiative transfer model. Figure 11a compares the influences of aerosols (red), altitude (green), and water vapor (blue) on spectral differences in Antofagasta and on the Chajnantor Plateau. Each curve in Fig. 11a was computed by calculating the ratio between a spectrum modeled based on the conditions observed in Antofagasta on 08.02.2015, the “baseline” spectrum, and a “perturbed” spectrum computed with some parameters varied, while the rest were held constant. For example, to determine the influence of aerosols, the (extremely low) aerosol loading observed on the Chajnantor Plateau on the 02.03.2015 was used to compute the perturbed spectrum while all other parameters were held constant. Therefore, the red shaded area in Fig. 11a indicates the contribution of aerosols to the observed difference between the spectra in Antofagasta and on the Chajnantor Plateau. To estimate the influence of altitude, the perturbed spectrum was computed using both the aerosol loading and the altitude (5100 m) observed on the Chajnantor Plateau on 02.03.2015; therefore, the green shaded area indicates the contribution of changes in the optical path to the observed difference between the spectra in Antofagasta and on the Chajnantor Plateau. Finally, the perturbed spectrum was computed with the aerosol loading, the altitude, and the very low PW value (2 mm) observed on the Chajnantor Plateau on 02.03.2015; therefore, the blue shaded area in Fig. 11a indicates the contribution of the PW to the observed difference between the spectra in Antofagasta and on the Chajnantor Plateau.

The influences described above are quantified in terms of total irradiance as follows. The difference between total irradiances in Antofagasta and on the Chajnantor Plateau was 15% (1143 W/m2 versus 990 W/m2, at SZA = 20°). By using the radiative transfer model, we found that PW accounts for 9% of that difference (computed by comparing spectra computed with PW = 2 mm and with PW = 35 mm), while aerosols account only for about 2%. In the IR range (wavelengths longer than 700 nm), PW accounts for 18% of the difference of about 25% between IR irradiances in Antofagasta and on the Chajnantor Plateau, while aerosols (AOD = 0.015 versus AOD = 0.12 and SSA = 0.98 versus SSA = 0.90) account only for about 1.3%. The role of aerosols is more important at shorter wavelengths. In the visible range (400–700 nm) for example, aerosols account for nearly half (2.7%) of the difference of about 6% between visible irradiances in Antofagasta and on the Chajnantor Plateau. In the UV-A range (315–400 nm), aerosols explain 5% of the difference of about 16% between the UV-A irradiances in Antofagasta and on the Chajnantor Plateau. In the UV-B range, aerosols account for 6.5% of the difference of about 31% between the UV-B irradiances in Antofagasta and on the Chajnantor Plateau. Note that the role of the ozone was negligible since the TOC was nearly the same in Antofagasta and on the Chajnantor Plateau at the moment of the measurements.

In order to compute Fig. 11b, we applied exactly the same sequence described in the case of Fig. 11a, but using the parameters corresponding to Calama instead of those corresponding to the Chajnantor Plateau. The difference between the total irradiances in Antofagasta and in Calama was 8% (1071 W/m2 versus 990 W/m2, at SZA = 20°). By using the radiative transfer model, we found that the PW accounts for 4.7% of that difference (computed by comparing spectra computed with PW = 11 mm and with PW = 35 mm), while aerosols (AOD = 0.015 versus AOD = 0.05 and SSA = 0.98 versus SSA = 0.94) account only for about 1.5%. In the IR range, PW accounts for 10% of the difference of about 14% between IR irradiances in Antofagasta and in Calama, while aerosols account only for about 1%. In the visible range, aerosols account for about 1.8% of the difference of about 3.7% between visible irradiances in Antofagasta and in Calama. In the UV-A range, aerosols explain 3.4% of the difference of about 8.7% between the UV-A irradiances in Antofagasta and in Calama. In the UV-B range, aerosols account for 4.5% of the difference of about 16% between the UV-B irradiances in Antofagasta and in Calama. Again, note that the role of the ozone was negligible since the TOC was nearly the same in Calama and on the Chajnantor Plateau at the moment of the measurements.

Since the blue curve in Fig. 11a is very close to the blue curve in Fig. 10a, we conclude that the contributions of the different aerosol loading (red area), the shorter optical path length (green area), and the different water vapor column (blue area), explains most the differences detected between the spectra in Antofagasta and on the Chajnantor Plateau. A similar conclusion arises in the case of Antofagasta and Calama by comparing Figs 10b and 11b.

## Summary and Conclusions

Aimed at the characterization of the spectral irradiance, we carried out ground-based measurements at seven locations in the Atacama Desert that ranged from the city of Antofagasta (on the southern pacific coastline) to the Chajnantor Plateau (5,100 m altitude). Our spectral measurements allowed us to retrieve the TOC, PW, AOD, and SSA values at each location.

Compared with locations in the northern hemisphere, the Atacama Desert is an area characterized by relatively low TOC values. We confirmed that the ozone column in the Atacama Desert can be in February 50–60 DU lower than the TOC value in June at similar latitudes in the northern hemisphere. These differences in the TOC contribute significantly to interhemispherical differences in the UV-B irradiance (calculated by integrating the spectra over the interval 290–315 nm). Discarding other effects, we found that due to the different TOC value, the UV-B irradiance measured in Calama in February was about 20% higher than that measured under similar conditions in Izaña (Tenerife) in June. Although the UV-B irradiance represents a very small fraction (0.2–0.3%) of the total irradiance, the relatively high UV-B irradiance in the Atacama Desert require careful quantification due to potential adverse effects on the biosphere and on the degradation of PV technologies.

The Atacama Desert is generally considered a zone of low PW values. Although the water vapor column measured in Antofagasta (on the southern pacific coastline) was high (peaked at 35 mm during the campaign), we did find low PW values at higher locations, including extremely low values (<1 mm) on the Chajnantor Plateau. Changes in the water vapor column were found to significantly contribute to the increase observed in the IR irradiance with the altitude; differences of about 27% in the IR irradiance (calculated by integrating the spectra at wavelengths longer that 700 nm) were found between Chajnantor and Antofagasta.

Compared with values measured at desert sites in northern Africa for example, the AOD at 550 nm is relatively low; we measured values lower than 0.12 at all locations in the Atacama Desert during the campaign. We also confirmed that the fraction of absorbing aerosols in the Atacama Desert is significantly lower (SSA > 0.9) than at locations heavily affected by urban pollution (for example, Santiago de Chile; SSA ~ 0.7). Discarding other effects, we found that due to the different aerosol load, the irradiance measured in D. de Almagro was higher (about 2.5% in the IR range, 5% in the visible range, 8% in the UV-A range, and up to 10% in the UV-B range) than that measured under similar conditions in Santiago de Chile. Aerosols also contributed to the increases in the irradiance with the altitude. Extremely low AOD values were measured at high-altitude locations (for example, on the Chajnantor Plateau; AOD ~ 0.015).

We found that differences in the PW, AOD, and SSA values (as well as the shorter optical path length at high-altitude locations) lead to increases in the surface irradiance with altitude in the Atacama Desert. The low PW values at high-altitude locations drove the increases with the altitude observed in the surface IR irradiance. In the visible range, reductions in the aerosols loading with the altitude explained nearly half of the increment observed in the surface irradiance with the elevation. The rest of the difference arose from the shorter optical path length (through scatterers in the atmosphere), which also accounted for most of the increment in the surface UV irradiance with the altitude. The effect of the ozone in the increment of the surface UV with the altitude in the Atacama Desert was small since the TOC was nearly the same at all locations during the campaign.

Although these figures should not be taken as general, we estimate that in the range 0–2500 m altitude, surface irradiance increases with the altitude by about 5.5% per km in the infrared range, 1.5% per km in the visible range, 4% per km in the UV-A range, and 9% per km in the UV-B range. In the range 2500–5000 m altitude, surface irradiance increases with the altitude by about 3.5% per km in the IR range, 0.7% per km in the visible range, 2% per km in the UV-A range, and 4% by km in the UV-B range. These changes in the spectra with the altitude lead to an increment in the total irradiance of about 3.5% per km in the range 0–2500 m altitude, and about 2% per km in the range 2500–5000 m altitude.

## Additional Information

**How to cite this article**: Cordero, R. R. *et al*. The Solar Spectrum in the Atacama Desert. *Sci. Rep.* **6**, 22457; doi: 10.1038/srep22457 (2016).

## Figures and Tables

**Table 1:** Locations and sky conditions during the campaign.

Location	Altitude	Position	Date	Sky Conditions
Antofagasta	114 m	23°27'16.07′′S 70°26'21.40′′W	08.02.2015	Cloudless
			09.02.2015	Stratocumulus clouds in the Morning
			10.02.2015	Cloudless
Caldera	208 m	27°15'53.96′′S 70°46'32.65′′W	12.02.2015	Cloudless
			13.02.2015	Stratocumulus clouds in the Morning
			14.02.2015	Stratus clouds
El Salvador	1600 m	26°18'50.88′′S69°45'10.71 W	16.02.2015	Cloudless
			17.02.2015	Cloudless
			18.02.2015	Cloudless
D. de Almagro	780 m	26°23'40.74′′S70° 3'2.40′′W	20.02.2015	Cloudless
			21.02.2015	Cloudless
			22.02.2015	Cloudless
Calama	2293 m	22°29'50.84′′S68°54'51.57′′W	24.02.2015	Cloudless
			25.02.2015	Cloudless
			26.02.2015	Cloudless
			27.02.2015	Altostratus Clouds
Chajnantor	5100 m	22° 57′ 30″ S 67° 47′ 10″ W	02.03.2015	Cloudless
			03.03.2015	Cirrus clouds in the Afternoon
			04.03.2015	Cirrus clouds in the Morning
M. Elena	1184 m	22°16'28.64′′S 69°33'59.08′′W	07.03.2015	Cloudless
			08.03.2015	Cloudless
			09.03.2015	Cloudless

**Table 2:** Increases in the irradiance with the altitude estimated from our ground-based measurements.

	Altitude Range	
	0–2500 m	2500–5000 m
UV-B Irradiance	+9%/km	+4%/km
UV-A Irradiance	+4%/km	+2%/km
UV Irradiance	+4%/km	+2%/km
Visible Irradiance	+1.5%/km	+0.7%/km
IR Irradiance	+5.5%/km	+3.5%/km
Total Irradiance	+3.5%/km	+2%/km
